# National Hockey League Fights per Game and Viewership Trends: 2000–2020

**DOI:** 10.3389/fspor.2022.890429

**Published:** 2022-06-30

**Authors:** Thomas A. Fortney, Liana J. Tedesco, Nathan J. Kopydlowski, Jack F. Korzelius, Sohil S. Desai, Charles A. Popkin

**Affiliations:** ^1^Columbia University Irving Medical Center, New York City, NY, United States; ^2^USA Hockey Team Physician and Member of the USA Hockey Safety and Protective Equipment Committee (SPEC), Colorado Springs, CO, United States

**Keywords:** fighting, hockey, National Hockey League (NHL), concussion, ice hockey injuries

## Abstract

**Background:**

Though once considered an integral part of professional hockey, fighting carries significant health risks to players. Fighting has remained legal in the National Hockey League (NHL) due to its purported economic and entertainment value. However, fights per game have diminished over the past 20 years, challenging the necessity of fighting to promote fan attendance.

**Hypothesis:**

Despite decreasing fighting rates, attendance has been stable and is negatively associated with fights per game.

**Methods:**

Two public databases were reviewed to determine attendance, fighting majors, goals scored, and games played for each NHL team from 2000 to 2020 and averaged on a per game basis. Univariate analysis was used to evaluate relationships between attendance and fights, attendance and goals, as well as goals and fights.

**Results:**

Fights per game decreased from a peak of 0.64 in 2002 to a low of 0.18 in 2020, while average attendance increased from a low of 16,549 in 2004 to a peak of 17,768 in 2013, before settling between 17,400 and 17,500 during the final three seasons of the study period. A significant negative correlation was found between attendance and fights per game (*R* = −0.6617, *p* = 0.0020). There was a positive, but not significant correlation between attendance and goals per game (*R* = 0.2457, *p* = 0.3105). A significant inverse correlation existed between goals per game and fights per game (*R* = −0.521, *p* = 0.0222).

**Conclusions:**

NHL fighting rates have diminished during the past two decades, while fan attendance has increased. A significant negative correlation exists between fan attendance and fights per game, casting doubt on fighting's entertainment value. Meanwhile, a significant inverse correlation was noted between goals per game and fights per game. Taken together, these findings suggest fans may prefer higher scoring and less violent competitions. We conclude by suggesting that prohibiting fights in the NHL could improve player safety without negatively impacting fan attendance.

## Introduction

Ice hockey is a fast-paced, collision sport punctuated by interludes of fighting and is among the most popular sporting events in North America (Mathewson, 2019). Historically, fighting has been considered a cornerstone of professional ice hockey with purported economic and entertainment value (Haisken-DeNew and Vorrell, 2008). Proponents also argue fighting has a role in regulating the tempo of the game and deterring other acts of violence on the ice (Bernstein, 2006; Hardy and Holman, 2018). However, leniency toward fighting in hockey can lead to a variety of injuries including concussions, oculofacial trauma and hand fractures (Juhn et al., 2002; Kuhn and Solomon, 2015). In recent years, several notable National Hockey League (NHL) “enforcers” have been diagnosed with chronic traumatic encephalopathy (CTE) on post-mortem autopsies, demonstrating the potential long-term health consequences of fighting in hockey (McKee et al., 2013; Prewitt, 2018).

According to the 2021 NHL rulebook, players who participate in a fight are reprimanded with a five-minute major penalty but are not ejected from the game. If one player is deemed the instigator of the fight, that player will receive an additional 2-min minor and 10-min misconduct, but is then allowed to return to play (Goldschmied and Espindola, 2013). In contrast, fighting is punished with a game misconduct penalty in nearly all European professional hockey leagues, and the offending players are barred from returning to the game (Jääkiekon Kansainvälinen Sääntökirja 2021–2022, 2021; KHL Disciplinary Regulations, 2021; Pravidla Ledního Hokeje 2021/2022, 2021; Spelregler för Elitishockey 2021–2022, 2021; IIHF Official Rule Book, 2021/22; Neue Regeln Ab Der Saison, 2021/22). As a result, fighting is exceedingly rare in European hockey games (Bernstein, 2006). Outside of boxing and martial arts, the NHL is the only professional enterprise in North America that allows fighting as part of competition (Bernstein, 2006; Smith et al., 2011; Plassche et al., 2022).

In spite of historic arguments for the importance of fighting in hockey, the number of fighting majors per game has steadily decreased over the last 20 years in the NHL (Bernstein, 2006). The goal of this study was to determine if declining rates of fighting have impacted NHL fan attendance. We hypothesize that despite decreasing rates of fighting in the NHL during the last two decades, viewership trends have been stable or even increasing during that time. Furthermore, if fan attendance has not been affected by diminished rates of fighting, this would call into question the economic and entertainment value of fighting and its role in the modern-day NHL.

## Methods

Two publicly available internet databases [hockeydb.com (National Hockey League History and Statistics) and hockeyfights.com (NHL Team Fighting Majors Leaders)] were reviewed to determine the total fan attendance, fighting major penalties, goals scored and games played for each NHL team during the years of 2000–2020. A “major” penalty requires that the offending player spend 5 min in the penalty bench, as compared to a “minor” penalty for which the offending player is ruled off the ice for 2 min (National Hockey League Official Rules, 2020–2021). Fan attendance, fighting majors, and goals scored were averaged on a per game basis for each NHL season during the study period. To allow for historical comparison, total fighting majors and games played were recorded from 1953 to 2020, as fighting majors were not officially recorded prior to the 1953–1954 season. Average fighting majors per game were calculated from 1953 to 2020.

Table 1 lists data from the 2000–2001 season through the 2019–2020 season. It should be noted that the entire 2004–2005 NHL season was canceled due to a lockout. Additionally, the total number of games played in the 2019–2020 season was reduced due to game cancellations during the COVID-19 pandemic. Meanwhile, the number of games per season increased in 2017–2018 with the addition of the Vegas Golden Knights expansion team. For this reason, we used average attendance, fights per game, and goals per game in our statistical analysis.

**Table 1:** Games played, fighting majors, fan attendance, and goals scored during 2000–2020 NHL season.

**Years**	**Season**	**Games played**	**Total fighting**	**Total**	**Total goals**	**Attendance**	**Fights**	**Goals**
			**majors**	**Attendance**	**scored**	**per game**	**per game**	**per game**
2000–2001	2001	2,460	1,354	40,734,730	6,790	16,559	0.55	2.76
2001–2002	2002	2,460	1,586	41,225,910	6,445	16,759	0.64	2.62
2002–2003	2003	2,460	1,322	40,811,810	6,519	16,590	0.54	2.65
2003–2004	2004	2,460	1,562	40,711,688	6,322	16,549	0.63	2.57
2004–2005^*^	2005	–	–	–	–	–	–	–
2005–2006	2006	2,460	918	41,707,414	7,577	16,954	0.37	3.08
2006–2007	2007	2,460	987	41,722,666	7,257	16,960	0.40	2.95
2007–2008	2008	2,460	1,316	42,576,450	6,839	17,308	0.53	2.78
2008–2009	2009	2,460	1,458	42,989,894	7,159	17,476	0.59	2.91
2009–2010	2010	2,460	1,423	41,997,776	6,986	17,072	0.58	2.84
2010–2011	2011	2,460	1,284	42,120,940	6,863	17,122	0.52	2.79
2011–2012	2012	2,460	1,089	42,936,348	6,716	17,454	0.44	2.73
2012–2013	2013	2,460	692	43,708,460	6,691	17,768	0.28	2.72
2013–2014	2014	2,460	933	43,516,990	6,740	17,690	0.38	2.74
2014–2015	2015	2,460	780	43,066,974	6,716	17,507	0.32	2.73
2015–2016	2016	2,460	687	43,226,792	6,667	17,572	0.28	2.71
2016–2017	2017	2,460	745	43,090,016	6,814	17,516	0.30	2.77
2017–2018†	2018	2,542	557	44,348,880	7,550	17,446	0.22	2.97
2018–2019	2019	2,542	450	44,373,644	7,651	17,456	0.18	3.01
2019–2020%	2020	2,164	388	37,695,623	6,535	17,419	0.18	3.02

* *2004-2005 season cancelled due to NHL labor lockout. ^†^Vegas Golden Knights became the 31st NHL team in 2017-2018, increasing the total league games played to 2542 % 2019-2020 season shortened due to COVID-19 global pandemic*.

Univariate analysis was used to evaluate for relationships between attendance and fights per game, attendance and goals scored per game, as well as goals scored and fights per game. An additional univariate analysis was performed to compare attendance and total fighting majors. The Shapiro-Wilk test was used to examine the distribution of individual variables for normality. Data that was normally distributed was presented as mean and standard deviation. Pearson product-moment correlation test was performed when comparing two continuous variables. Statistical significance was set at *p* < 0.05. All analyses were performed with R Version 4.1.0 (R Foundation for Statistical Computation, Vienna, Austria).

## Results

**Figure 1:**
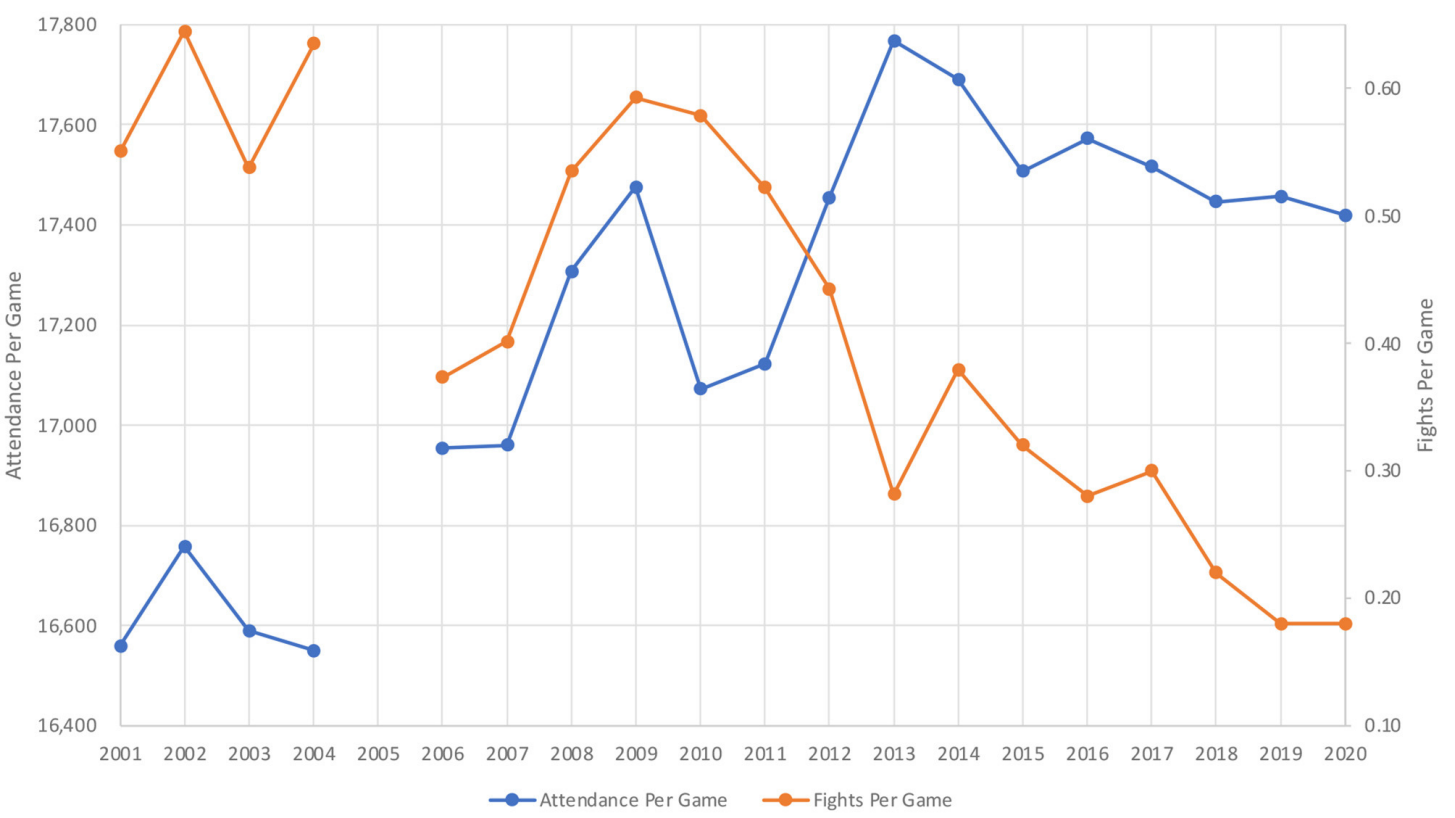
Graph comparing rates attendance and fighting in the NHL during the period of 2000–2020. Average attendance per game is reported on the first *Y-*axis while the number of fights per game is denoted on the second *Y-*axis. The NHL season is listed on the X-axis with the year representing when the Stanley Cup championship was held for each season. For example, the 2000–2001 NHL season is listed as 2001, as the Stanley Cup championship for that season was held in the spring of 2001. This plot demonstrates the trend of decreasing fighting rates and increasing attendance during the last 20 years. Note the entire 2005 season was canceled due to a lock-out.

Attendance per game (17,768) was greatest during the 2012–2013 season, while total league attendance (44,373,644) was highest during the 2018–2019, though this was after the addition of an expansion team (Table 1). Excluding the shortened 2019–2020 season, total attendance (40,711,688) and attendance per game (16,549) were lowest during the 2003–2004 season (Figure 1). Total fighting majors (1,586) and fights per game (0.64) each reached a peak in 2001–2002. Conversely, total fighting majors (450) and fights per game (0.18) reached a nadir in 2018–2019, excluding the shortened 2019–2020.

Total goals scored (7,577) and goals per game (3.08) were highest during the 2005–2006 season, immediately following the 2004–2005 lockout (Table 1). Meanwhile the fewest total goals (6,322) and goals per game (2.57) were registered during the 2003–2004 season, just prior to the lockout. There was a trend of increasing goals per game during the most recent three seasons of the study period (2018–2020), with all seasons averaging 2.97 goals per game or greater.

During the last two decades, there was a statistically significant negative correlation between attendance and fights per game (*R* = −0.6617, *p* = 0.0020). Likewise, comparing attendance and total fighting majors resulted in statistically significant negative correlation (*R* = −0.665, *p* = 0.0019). When comparing attendance and goals per game, there was a positive correlation, though not statistically significant. (R = 0.2457, *p* = 0.3105). Finally, there was statistically significant inverse correlation between goals per game and fights per game (*R* = −0.521, *p* = 0.0222).

## Discussion

Fighting has long been a part of professional hockey in North America, and its role in the sport has evolved over the last century. The original rules of ice hockey, penned in Montreal, Canada in 1877, did not prescribe any penalty for fighting (Bernstein, 2006). It would be another 45 years before Rule 56 was introduced to the NHL, which punished players who engaged in “fisticuffs” with a 5-minute penalty (Hardy and Holman, 2018). Despite this addition, the rates of fighting increased over the ensuing decades reaching an all-time high in the 1980s (Bernstein, 2006; NHL Team Fighting Majors Leaders). In 1992, the NHL implemented a rule dictating that the instigator of a fight would receive a game misconduct penalty (Goldschmied and Espindola, 2013). Current NHL rules state that players who participate in a fight will be assessed a 5-minute major penalty, and an instigator of a fight will receive an additional 2-min minor and 10-min misconduct (Goldschmied and Espindola, 2013). Unlike most European professional leagues (Table 2), NHL players are not ejected from the game following a fight (IIHF Regelbuch; National Hockey League Official Rules, 2020–2021; Jääkiekon Kansainvälinen Sääntökirja 2021–2022, 2021; Pravidla Ledního Hokeje 2021/2022, 2021; Spelregler för Elitishockey 2021–2022, 2021; IIHF Official Rule Book, 2021/22).

**Table 2:** Comparison of penalties for fighting among top professional ice hockey leagues in North America and Europe.

**League name (abbreviation)**	**Country**	**Penalty time assessed (minutes)**	**Major penalty (Y/N)**	**Game misconduct (Y/N)**	**Instigator penalty (Y/N)**	**Instigator penalty time assessed**	**Enforce IIHF rules**
American Hockey League (AHL)	United States / Canada	5	Y	N	Y	2 min minor + 5 min major + 10 min misconduct	N
Czech Extraliga (ELH)	Czech Republic	5	Y	Y	Y	2 min minor + 5 min major + Game misconduct	Y
Deutsche Eishockey Liga (DEL)	Germany	5	Y	N^*^	Y	5 min major + Game misconduct	N
Kontinental Hockey League (KHL)	Russia	5	Y	N	Y	2 min minor + 5 min major + 10 min misconduct	N
Liiga	Finland	5	Y	Y	Y	2 min minor + 5 min major + Game misconduct	Y
National League (NL)	Switzerland	5	Y	Y	Y	2 min minor + 5 min major + Game misconduct	Y
National Hockey League (NHL)	United States / Canada	5	Y	N	Y	2 min minor + 5 min major + 10 min misconduct	N
Swedish Hockey League (SHL)	Sweden	5	Y	Y	Y	2 min minor + 5 min major + Game misconduct	Y

* *After 3 fighting major penalties during 1 season, player is suspended 1 game*.

*Y, yes*.

*N, no*.

*IIHF, International Ice Hockey Federation*.

Supporters of fighting in hockey cite its economic and entertainment value, along with its role in “self-policing” the game. This theory holds that the threat of a fight with an opposing “enforcer” deters players from taking harmful actions against the other team, such as slashing, roughing, verbal abuse or illegal body checks (Bernstein, 2006). Opponents of fighting note the numerous health risks to players and the scarcity of fighting in European professional hockey. With the notable decrease in fighting rates over the last 20 years, it is time to reevaluate the role of fighting in the modern-day NHL. This study sought to determine if diminishing rates of fighting in the NHL impacted attendance and provide evidence against the historic argument that fighting is necessary for economic or entertainment value.

As previously noted, a statistically significant negative correlation was found between attendance and fights per game (*R* = −0.6617, *p* = 0.0020). There was an inverse correlation between goals per game and fights per game that reached statistical significance (*R* = −0.521, *p* = 0.0222). There was a positive correlation, though not statistically significant, between attendance and goals per game (R = 0.2457, *p* = 0.3105). This data suggests that NHL fans prefer games with lower fighting and higher scoring, though there may be other factors affecting fans' decision to attend games.

Proponents of fighting in hockey argue that it serves as a means of self-regulating player conduct and safety. Some players feel that the instigator rule has impeded the enforcer's ability to regulate the game, because the 2-min instigator penalty would put the enforcer's team at a disadvantage (Bernstein, 2006). However, arguments that fighting helps to self-govern the game are theoretical and difficult to quantify. Supporters also argue that fighting is a method to modulate momentum during a game (Bernstein, 2006; Leard and Doyle, 2011). Enforcers may challenge opponents to a fight when their team is losing, with the notion that winning the fight will shift momentum to their side. However, a study examining the effects of momentum and fighting on winning in the NHL found that winning fights did not translate to winning more games (Leard and Doyle, 2011).

**Figure 2:**
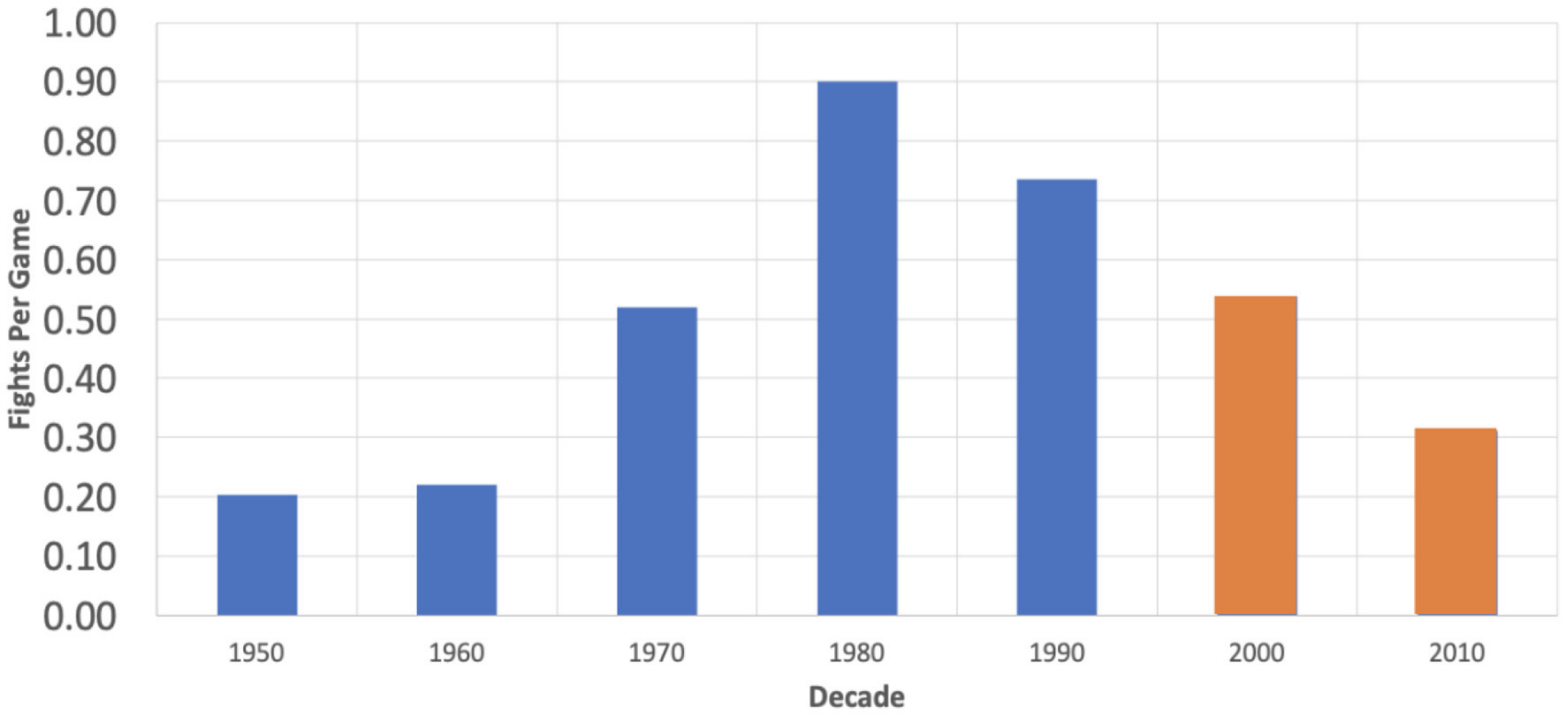
Graph and table reporting the average fights per game by decade in the NHL.

Additional arguments for the continuation of fighting in hockey relate to its potential economic and entertainment value. Jones et al. evaluated the relationship between hockey violence and game attendance during the 1989–1990 season, finding a positive correlation in American cities, but not Canadian (Jones et al., 1996). Notably, the Jones study reviewed data from an era with much higher fighting rates (Figure 2) and our study examining data from the past 20 seasons found a significant negative correlation between attendance and fights per game (*R* = −0.6617, *p* = 0.0020).

**Figure 3:**
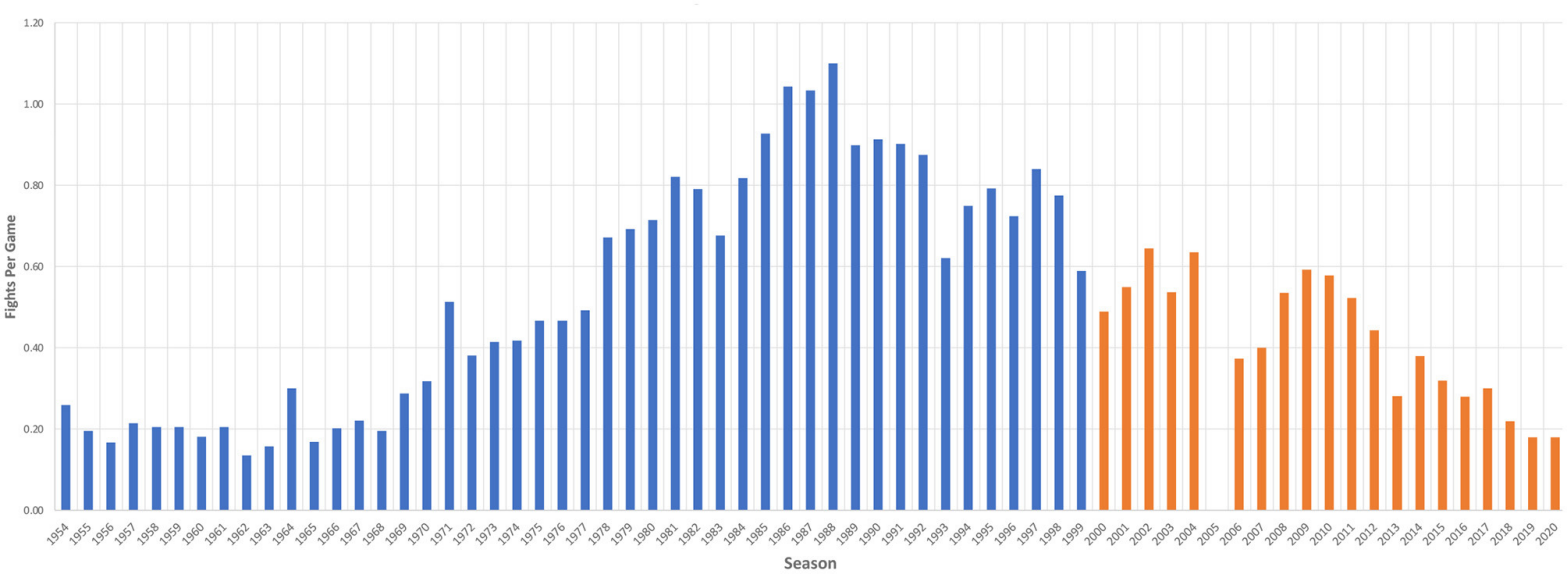
Graph demonstrating historical rates of fighting in the NHL from 1953 to 2020. This figure demonstrates average fights per game from the 1953 to 2020 seasons. Only the average value was included as the number of games played in each season was dependent on the number of NHL teams and length of the season, which has varied over the decades.

To add historical perspective, the highest fights per game average (1.10) was recorded during the 1987–1988 season, while the lowest fights per game average (0.14) occurred during the 1961–1962 season (Figure 3).

The relationship between fighting and fan attendance in the NHL has changed over the last several decades. Studies ranging from 1984 to 2003 found that higher fighting rates correlated with greater attendance at NHL games (Jones, 1984; Jones et al., 1993, 1996; Paul, 2003). While literature as recent as 2011 demonstrated that fighting decreased the likelihood of winning games in the NHL, fighting was still associated with increased fan attendance (Stewart et al., 1992; Coates et al., 2011). In 2016, an economic model for optimizing NHL club profitability found that fighting had a negative effect on attendance and did not promote profit-maximizing (Rockerbie, 2016). Our current study provides further evidence that fighting is now negatively correlated with attendance during NHL competitions.

A 2008 study found that an NHL player could earn a wage premium for fighting. Though no monetary bonuses were paid to players for fighting, the authors found that a player's yearly salary increased on average by $18,135 per fight won and $11,993 per fight lost, however, it is unclear what other factors may have contributed to these increases (Haisken-DeNew and Vorrell, 2008). However, this study was conducted using data from the 1996–2007 seasons when fighting rates were higher, and it is unclear what the wage premium is for a fight in the modern NHL.

Critics of hockey fights cite the risk of injury to players including hand fractures, oculofacial trauma, concussions and CTE (Donaldson et al., 2013; Donaldson et al., 2014; Hutchison, 2015; Kuhn and Solomon, 2015; Smith et al., 2019a,b; Plassche et al., 2022). Head injuries are common among NHL players, and approximately 9% of all concussions result from fighting (Donaldson et al., 2013; Hutchison, 2015). Concussions contribute significantly to games missed and account for salary loss of $42.8 million annually (Depken et al., 2020). There is growing awareness surrounding the sequelae of head trauma and concussions in athletes (McKee et al., 2013; Kuhn and Solomon, 2015; Smith et al., 2019b). CTE is a clinical syndrome marked by depression, irritability, aggression, short-term memory loss and increased suicidal ideation. These symptoms may appear 8–10 years after episodes of mild, repetitive brain trauma. More severe neurological manifestations appear in advanced stages and include dementia, gait disturbances, speech abnormalities and parkinsonism (McKee et al., 2013).

In 2011, three prominent NHL enforcers tragically passed away, two by suicide and one by drug overdose. All three players were subsequently diagnosed with CTE on postmortem autopsy, as were numerous other former NHL enforcers in the last decade (McKee et al., 2013; Romero, 2015). Over 300 former players participated in a class action lawsuit against the NHL over its management of concussions (McCann, 2018). The suit claims the NHL regarded fighting as a major attraction and revenue generator but disregarded the potential harm to its players. The NHL denied culpability, but the suit was eventually settled for $19 million in 2018 (McIndoe, 2017; McCann, 2018).

Movements to curb fighting in hockey have been developing over the last decade, with experts at the Mayo Clinic Ice Hockey Summit voting in 2011, 2013, and 2017 to eliminate fighting in an effort to prevent concussions (Smith et al., 2011, 2015, 2019a). Smith et al. reviewed the negative effects of fighting and head trauma in hockey players and advocated for the ban of fighting at all levels of organized hockey (Smith et al., 2019b). The authors recommended that NHL players who engage in fighting receive a five-minute major penalty and game misconduct (or ejection), which would align with International Ice Hockey Federation rules (IIHF Official Rule Book; Smith et al., 2019b). Collegiate hockey in the United States has gone one step further by assigning a one-game suspension to any player who participates in a fight (Piotrowski, 2020). As a result, the National Collegiate Athletic Association reported only 20 fights over a 4-year period, compared to 3,091 NHL fights over the same duration. Smith et al. also noted an impediment to diagnosing concussions after a fight because the involved players are sent directly to the penalty box. Notably, this contradicts the NHL Concussion Policy which dictates that players who experience head trauma should be placed in a “quiet room” for concussion evaluation (Smith et al., 2019b).

There are several possible reasons for the decreased fighting rates over the last two decades. In a 2004 article, Depken and Wilson found that the addition of a second referee during the 1998 and 1999 NHL seasons, coincided with a reduction in fighting and increase in scoring. They also found that television viewership increased while in-arena attendance did not change during this time (Depken and Wilson, 2004; Depken et al., 2020). Following the 2004–2005 NHL lockout, several rules were implemented to reduce violence and increase goal scoring. Fighting was banned in the last 5 min of regulation time and in overtime. New emphasis was placed on the enforcement of interference, obstruction and hooking penalties, which benefited skilled players (Bernstein, 2006; National Hockey League Official Rules, 2020–2021). However, Depken et al. found that historically, fighting rates have decreased 4 to 5 years prior to any rule changes regarding fighting (e.g., instigator penalty and second referee). The authors suggest that reductions in fighting may have occurred in response to changes in community enforcement by the players, rather than deterrence from NHL rule changes. This also implies that the NHL rules committee may have been responding to changes in player behavior regarding fighting. Additionally, the authors propose that NHL players may have become more aware of the consequences of head trauma and concussions around this time, precipitating a drop in fighting rates (Depken et al., 2020).

The salary cap was also introduced in 2005 limiting the amount of money that could be spent on vital skilled players who often command higher salaries, leaving less money to pay enforcers (Haisken-DeNew and Vorrell, 2008). Another potential influence on fighting rates is the recent influx of European-trained players in the NHL. In 1980, European-born players accounted for just 9.1% of NHL players, but that number grew to 31.3% by the 2020 season (NHL Totals by Nationality-−1980–1981 Stats; (Podnieks). Given that fighting is illegal in nearly all levels of European competition, it is likely that the growing number of European players in the NHL have altered the rates of fighting.

This study is not without limitations. Firstly, the study period was limited to the past 20 NHL seasons because previous literature examining fighting rates in the 1980's and 1990's found a positive correlation between fighting and attendance (Jones, 1984; Jones et al., 1993, 1996; Paul, 2003). Fighting rates sharply decreased from 2000–2020, so we sought to determine how the decline in fighting rates impacted fan attendance. Secondly, while our study demonstrated a significant negative correlation between fights per game and attendance, we were not able to control for all variables that may affect fan attendance. Goals per game is another useful metric as the number of goals scored determines the outcome of the game and other studies have shown fans prefer higher scoring games (Paul and Chatt, 2011). Additionally, we did not examine other revenue sources such as TV viewership or merchandise, so it is possible that fan attendance is an imperfect proxy for viewership or revenue.

## Conclusion

Though once considered an integral part of professional ice hockey, fighting carries significant health risks to players. The number of fights per game in the NHL has steadily decreased during the past two decades, while fan attendance has increased. Our study demonstrated a statistically significant negative correlation between fan attendance and fights per game (*R* = −0.6617, *p* = 0.0020), while there was a positive correlation, though not statistically significant, between attendance and goals per game (R = 0.2457, *p* = 0.3105). There was a significant inverse correlation between goals per game and fights per game (*R* = −0.521, *p* = 0.0222). These results provide evidence that fighting is not necessary for economic or entertainment value and suggest NHL fans prefer higher scoring a less violent competitions.

## Data Availability Statement

The raw data supporting the conclusions of this article will be made available by the authors, without undue reservation.

## Author Contributions

All authors listed have made a substantial, direct, and intellectual contribution to the work and approved it for publication.

## Conflict of Interest

CP is a team physician for USA Hockey and a member of the USA Hockey Safety and Protective Equipment Committee. The remaining authors declare that the research was conducted in the absence of any commercial or financial relationships that could be construed as a potential conflict of interest.

## Publisher's Note

All claims expressed in this article are solely those of the authors and do not necessarily represent those of their affiliated organizations, or those of the publisher, the editors and the reviewers. Any product that may be evaluated in this article, or claim that may be made by its manufacturer, is not guaranteed or endorsed by the publisher.
